# Metformin potentiates rapamycin and cisplatin in gastric cancer in mice

**DOI:** 10.18632/oncotarget.3327

**Published:** 2015-02-28

**Authors:** Yu Yu, Wenzheng Fang, Tian Xia, Ying Chen, Yunshu Gao, Xiaodong Jiao, Suyun Huang, Jiejun Wang, Zhaosheng Li, Keping Xie

**Affiliations:** ^1^ Department of Medical Oncology, Changzheng Hospital, Shanghai 200070, China; ^2^ Department of Neurosurgery, The University of Texas MD Anderson Cancer Center, Houston, Texas 77030, USA; ^3^ Department of Gastroenterology, Changhai Hospital, Shanghai 200433, China; ^4^ Department of Pathology, Changhai Hospital, Shanghai 200433, China; ^5^ Department of Oncology, Qingdao, Shandong 266000, China; ^6^ Departments of Gastroenterology, Hepatology & Nutrition, The University of Texas MD Anderson Cancer Center, Houston, Texas 77030, USA; ^7^ Department of Oncology, Fuzhou General Hospital, Clinical Medical College of Fujian Medical University, Fuzhou, Fujian 350025, China

**Keywords:** gastric cancer, experimental therapy, metformin, mouse model, gene expression

## Abstract

Here we showed that pAMPKα and PTEN were down-regulated and p-mTOR, p-S6, p-4EBP1, MMP7, and DCN1 were up-regulated in human gastric cancer tissue samples as compared to that in the noncancerous tissues. Metformin inhibited tumor growth in mice. Also it enhanced cisplatin- or rapamycin-induced reduction of tumor growth as compared with treatment of either drug alone. In addition to activation of AMPK and suppression of the mTOR pathway, a series of increased and decreased genes expression were induced by metformin, including PTEN, MMP7, and FN1. We suggest that metformin could potentially be used for the treatment of gastric cancer especially in combination with cisplatin or rapamycin.

## INTRODUCTION

Gastric cancer is the second leading cause of cancer-related deaths worldwide and is clinically challenging, especially in East Asia [1]. Although its incidence has declined in the past several decades, gastric cancer is notorious for its ability to metastasize to regional lymph nodes, liver, and the peritoneal cavity. In addition, it often responds poorly to current therapeutic regimens and is frequently associated with a poor prognosis [2–5]. Therefore, understanding the underlying molecular aberrations and molecular prognostic markers in gastric cancer is critical to the design of effective therapeutics strategies.

The AMPK/mTOR signaling pathway has been widely studied in metabolic disorders and an increasing number of studies also suggest a potential role in cancer cell biology [6–10]. The AMP-activated protein kinase (AMPK) is a heterotrimeric serine/threonine kinase composed of a catalytic (α) subunit and two regulatory (β and γ) subunits. Upon energy stress, AMP directly binds to and activates the AMPKγ regulatory subunit and stimulates ATP production, leading to energy preservation for cell growth and proliferation. Following AMPK activation, multiple metabolic and signaling pathways are activated. In particular, AMPK regulates the mTOR signaling pathway, linking extracellular stimuli to intracellular signaling pathways involved in cell growth, proliferation and motility [11]. mTOR encompasses two distinct molecular complexes: mTORC1 and mTORC2. mTORC1 is activated by the PI3K/AKT pathway and regulates ribosomal biogenesis and protein synthesis by phosphorylating the downstream effectors, S6K1 and 4EBP1. Phosphorylated S6K1 in turn phosphorylates S6 (40S ribosomal protein S6), enhancing the translation of mRNAs, while phosphorylation of 4EBP1 suppresses its ability to act on downstream effectors. mTORC1 is inhibited by the TSC1/TSC2 complex. Activated AKT prevents TSC1/TSC2 complex formation, while activated AMPK stimulates TSC1/TSC2 complex thus creating a regulatory feedback loop [11]. In addition, AMPK can directly inhibit the mTORC1 pathway by stimulating the interaction of 14.3.3 and raptor proteins that can then suppress cell growth and biosynthetic processes under energy stress [12]. Aberrations of multiple elements of the mTOR pathway and their association with tumor progression have been extensively investigated in many types of cancers, making mTOR an appealing therapeutic target for cancer treatment [11].

Metformin is a first-line treatment for 2DM patients, while type 2DM and insulin resistance are found to be associated with the risk for development of several human solid cancers [13–15]. Moreover, prior studies have revealed that metformin lead to significant inhibition of cell proliferation and tumor growth. The AMPK system was a key target for metformin treatment. Activation of AMPK by metformin results in inhibition of mTOR signaling pathway and fatty acid synthesis (FAS), as well as stimulation of the p53/p21 axis [15, 16]. However, other potentially major mechanisms underlying metformin treatment for human gastric cancer remain unclear. This study was to evaluate the effect of metformin in a gastric tumor model system and to analyze its mechanism of action.

## RESULTS

### Metformin as a protective factor for gastric cancer patients with DM

Metformin can reduce the incidence and mortality for certain cancers, notably breast and colorectal carcinomas [15–18]. However, little is known about the impact of metformin use on gastric cancer. We used representative data-sets from Fuzhou General Hospital to assess whether metformin usage benefits gastric cancer patients with T2DM (Supplementary Table S1). Interestingly, metformin users had a median survival time of 63 months (95%CI: 52.6–73.9), which was significantly longer than that in non-metformin users (39 months; 95%CI: 30.9–47.3) (*P* = 0.028) (Supplementary Figure S1). However, metformin was not an independent prognostic factor in this small cohort (Supplementary Table S2).

### Expression patterns of genes associated with metformin-treatment in human gastric cancer tissues

**Figure 1:**
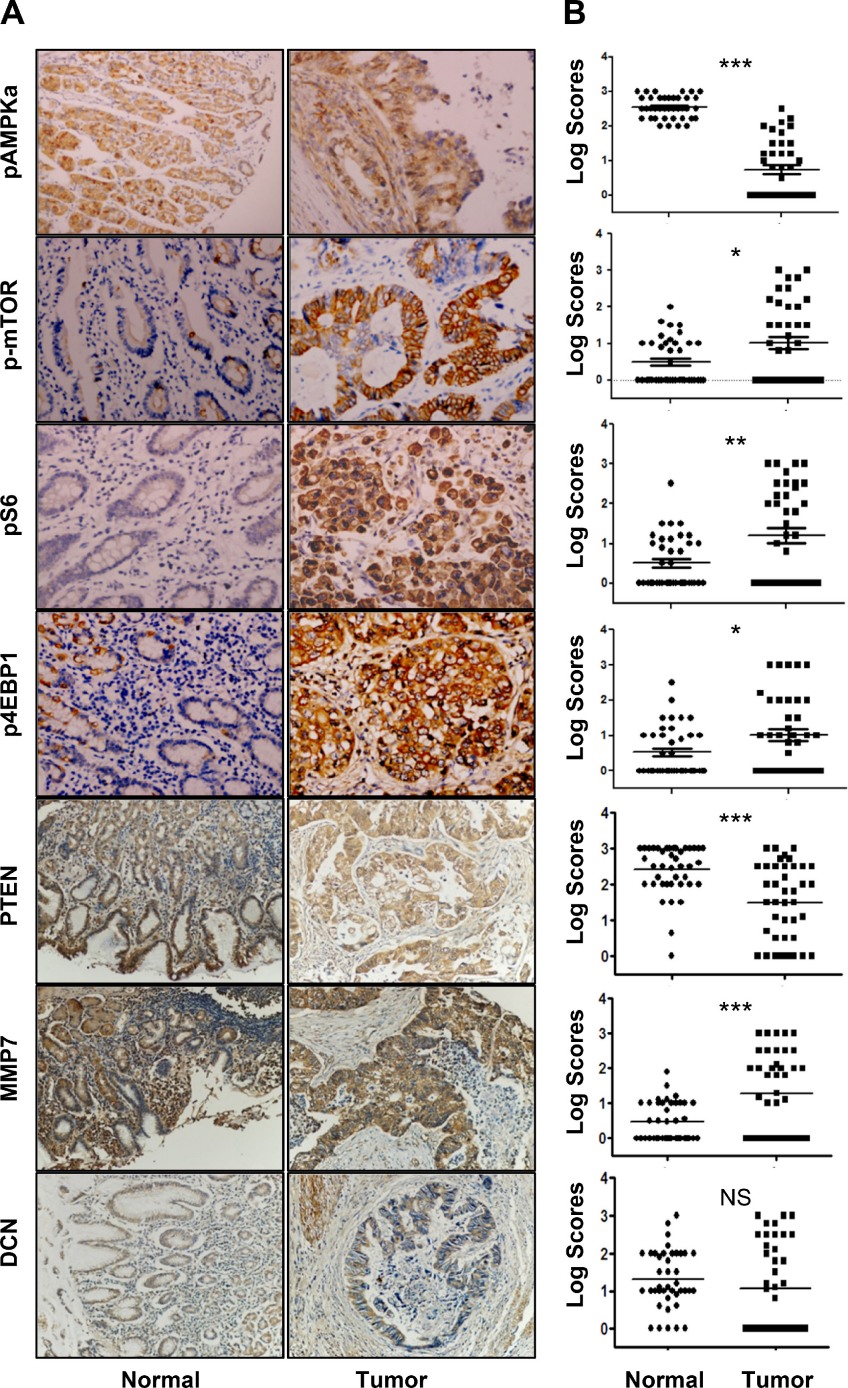
Expression profiles of pAMPKα, p-mTOR, pS6, p4E-BP1, PTEN, MMP7, and DCN in human gastric cancers and adjacent normal mucosa specimens **(A)** Representative staining of pAMPKα, p-mTOR, pS6, p4E-BP1, PTEN, MMP7, and DCN in non-neoplastic gastric mucosa (left panel) and gastric cancer tissues (right panel) (IHC × 200). **(B)** Graphical representation of the differences of pAMPKα, p-mTOR, pS6, p4E-BP1, PTEN, MMP7, and DCN staining in non-neoplastic gastric mucosa (N) and gastric cancer specimens (T)^*^P < 0.05; **P < 0.01, ***P< 0.001.

Protein expression levels of pAMPKα, p-mTOR, pS6, p4EBP1, MMP7, DCN and PTEN were analyzed in 39 resected primary gastric cancer samples. Expression of all those proteins was primarily in the plasma membrane or cytoplasm of normal gastric cells and tumor cells (Figure 1A). Expression of pAMPKα and PTEN in primary tumor tissue was significantly reduced as compared to that in adjacent noncancerous gastric tissue (Figure 1B). Expression of p-mTOR, pS6, p4EBP1, and MMP7 was elevated in primary tumor tissue as compared with that in adjacent noncancerous gastric tissue (Figure 1B). However, no significant difference of DCN expression was found between primary tumor tissue and adjacent noncancerous gastric tissue.

### Metformin inhibited gastric cancer cell proliferation and colony formation *in vitro* and growth in nude mice

**Figure 2:**
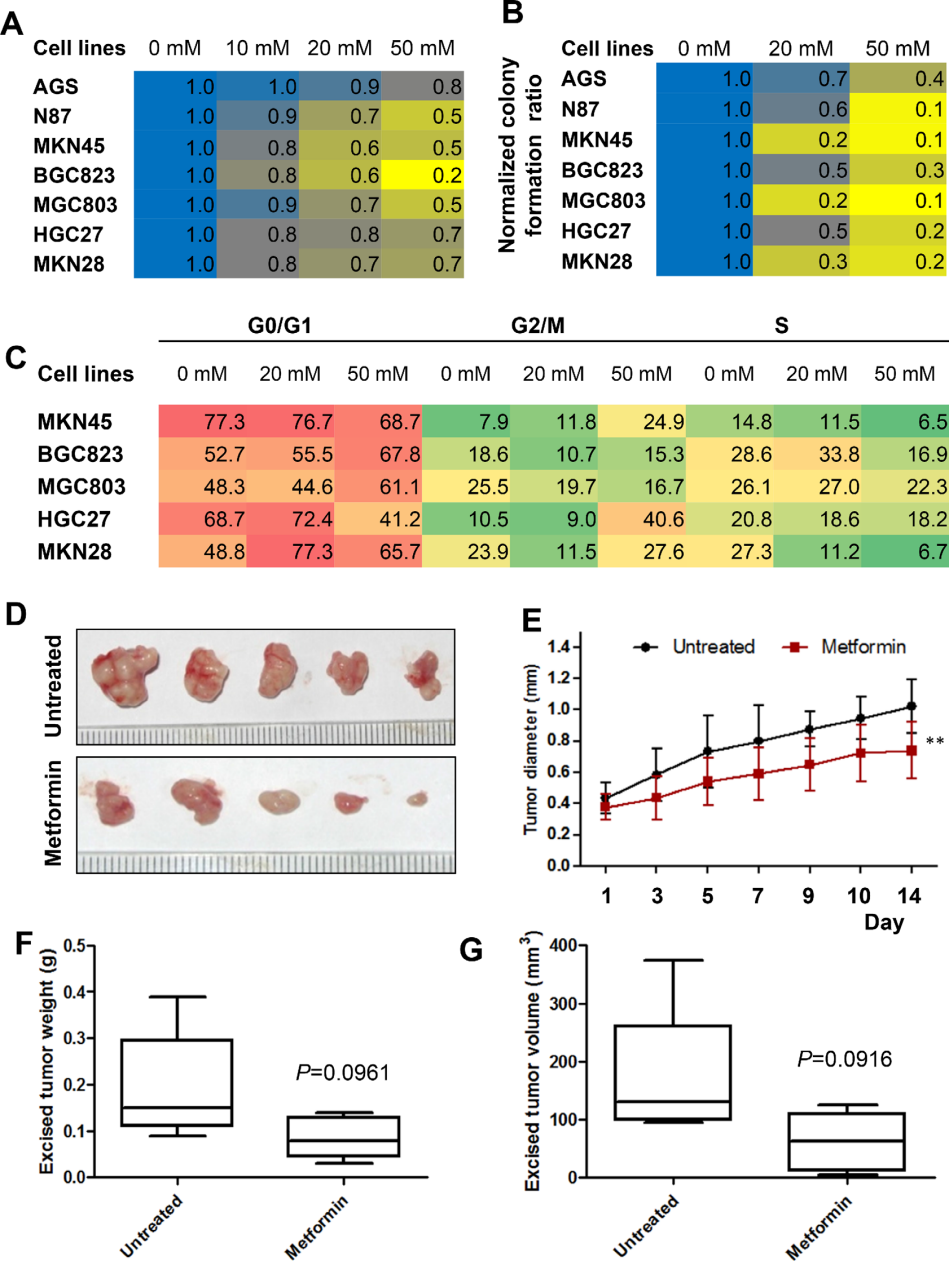
Metformin inhibited cell proliferation of gastric cancer (GC) cells *in vitro* and inhibits the growth of gastric cancer xenografts *in vivo* **(A)** GC cells were treated with metformin (0, 10, 20, and 50 mM) and a cell proliferation assay was performed at 0 h and 48 h. The results are expressed as percent of surviving cells at 48 h compared to that at 0 h. The result of 0 mM was designated as the calibrator and the normalized value for each concentration was divided by this calibrator. **(B)** GC cells were grown in 6-well plates and incubated with metformin (0, 10, 50 mM) for two weeks. The numbers of cell colonies (>50 cells) were calculated as: colonies/500 × 100. **(C)** GC cells were treated with metformin (0, 20, and 50 mM) and cell cycle analysis was performed at 48 h. **(D–G)** MKN45 cells (1 × 10^7^) were subcutaneously injected into the right flanks of female nude mice. When the tumors reached a mean diameter of 4 mm, the animals were treated with metformin (250 mg/kg) or NS (control) i.p. q.d. Tumor volumes were measured every two or three days. On day 15, mice were sacrificed and tumors were collected. **(D)** Representative images of the excised tumors of untreated and treated groups. **(E)** Longitudinal tumor growth curves of MKN45 cell xenografts after treatment. *P* < 0.01, *n* = 5/group. **(F)** Excised tumor weight of MKN45 cell xenografts (*P* = 0.0961). **(G)** Excised tumor volumes (*P* = 0.0916). Mid-point, mean; bars, SD (** < 0.01vs. control, Student's *t*-test).

To investigate the effects of metformin on cell proliferation, we used the CCK8 assay to measure AGS, N87, MKN28, MGC803, BGC823, HGC27, and MKN45 cell proliferation after treatment with metformin. Metformin significantly inhibited the proliferation rate of both gastric cancer cell lines in a dose-dependent manner (Figure 2A). Moreover, metformin significantly inhibited the rate of colony formation of gastric cancer cells in a dose-dependent manner (Figure 2B). The effects of metformin on cell cycle progression were further detected using flow cytometric analysis. As shown in Figure 2C, the proportion of cells in the S cell cycle phase was remarkedly decreased in metformin-treated cancer cells compared with untreated cells, especially in MKN45, BGC823 and MKN28 cell lines.

To determine whether metformin had inhibitory effect on cell proliferation *in vivo*, MKN45 cells were injected subcutaneously in nude mice and treated with metformin (250 mg/kg) or saline once daily by intraperitoneal injection once the tumors reached 4 mm in diameter. MKN45 xenograft tumor growth was significantly reduced with metformin treatment compared with untreated mice (Figure 2D & 2E). The mean weights and volumes of the excised tumors were approximately 47% and 52% less, respectively, in mice treated with metformin compared with untreated mice, although these differences were not statistically significant due to small number of mice enrolled (Figure 2F & 2G).

### Metformin enhances tumor growth inhibition in combination with cisplatin and rapamycin

**Figure 3:**
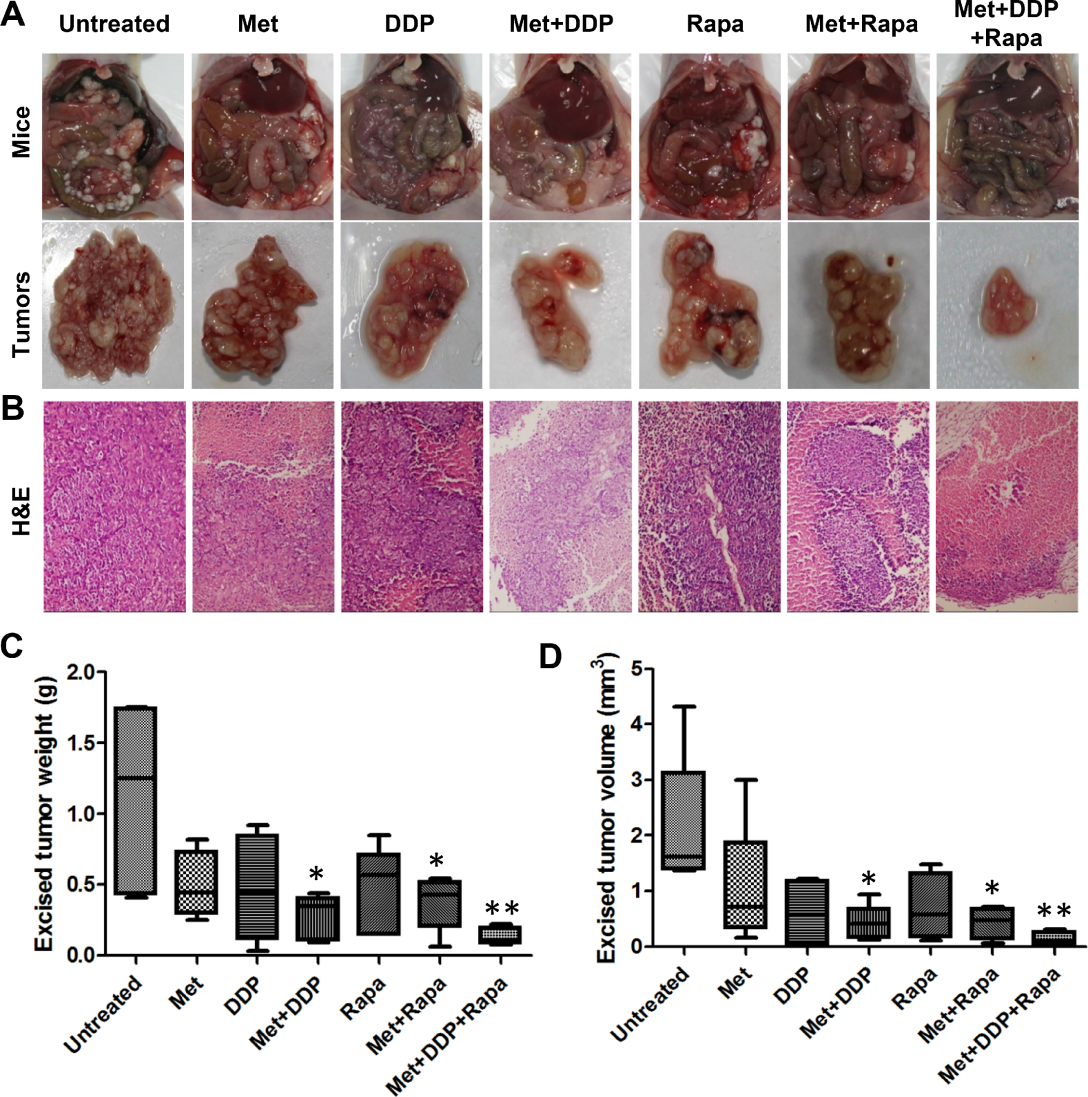
The combination of metformin with cisplatin (DDP) or rapamycin or both effectively inhibited peritoneal dissemination of gastric cancer **(A)**Representative gross morphology of vivisected mice showing peritoneal implanted tumors (top panel) and excised tumors (bottom panel) after two weeks of growth after treatment had begun (*n* = 5). **(B)** Representative images of H&E–stained tumor sections from gastric cancer xenografts (200×). **(C)** Excised tumor weights from each group with cumulative means; *P < 0.05; **P < 0.01, compared with the untreated group. **(D)** Excised tumor volume from each group with cumulative means; *P < 0.05; **P < 0.01, compared with the untreated group.

Given the fact that peritoneal dissemination is the most common metastatic pattern of gastric cancer and cisplatin (DDP) is one of the most effective chemotherapeutic drugs, we investigated whether the combination of metformin and cisplatin was more effective for tumor growth inhibition than either treatment alone using MKN45 xenografts. As shown in Figure 3A–3C, metformin (250 mg/kg) or cisplatin (4 mg/kg) treatment alone reduced tumor growth, but without significant difference from untreated mice. However, weekly treatments of cisplatin (4 mg/kg) along with continuous metformin (250 mg/kg) were more effective in inhibiting peritoneal tumor growth than either treatment alone. The combination significantly reduced the excised tumor weight and volume more than 50% compared with that of untreated mice (Figure 3B–3D). In addition, the abdominal circumference, representing the tumor burden carried in the peritoneum, was significantly less in the metformin and cisplatin-treated mice relative to control mice and was not significantly smaller in mice treated with metformin or cisplatin alone (Supplementary Figure S2A).

We have previously shown that rapamycin treatment alone resulted in inhibition of gastric cancer cell growth *in vitro* and *in vivo* [19]. Metformin and rapamycin treatment alone significantly inhibited cell proliferation of MKN45 cells *in vitro* (Supplementary Figure S2B). In peritoneal dissemination model, rapamycin alone remarkably reduced tumor growth, but showed no significant difference from the untreated group. However, the combination of rapamycin (2.5 mg/kg) and metformin (250 mg/kg) significantly reduced tumor growth as compared with untreated mice. The combination of the two drugs decreased tumor weight and volume more than 50% as compared with control mice (Figure 3A–3D). Similarly, the abdominal circumference was significantly less in metformin- and rapamycin-treated mice than in either treatment alone (Supplementary Figure S2A).

We next assessed tumor growth inhibition using a combination of all three drugs *in vivo*. Tumor weight and volume was reduced by ~90% in most of the mice and was significantly less than that in the untreated group (Figure 3C & 3D). Additionally, mice receiving the combination of treatments had the smallest abdominal circumference among all the groups. Histopathological analysis of peritoneal implanted tumor xenograft tissues indicate that mice treated with all three drugs exhibited significantly less tumor necrosis than that in other treatment groups (Figure 3B).

### Systemic side effect of treatment with metformin or in combination with cisplatin and/or rapamycin

To assess the physiologic impact of treatments, blood plasma was isolated from each treatment group and analyzed for hepatic function (AST and ALT), kidney function (creatinine and serum urea nitrogen), and glucose levels. In addition, we monitored animal weights throughout the course of the study and performed a histologic analysis of various organs. Weight loss was greatest in mice treated with regimens containing cisplatin, especially with cisplatin treatment alone. Significant weight loss was not observed in the other treatment groups. When cisplatin treatment was stopped, body weights recovered (Supplementary Figure S3). No obvious lesions were found in the tissue sections of gastric mucosa, colorectal mucosa, and kidney specimens from each group (Supplementary Figure S4). Similarly, no significant difference in kidney function or glucose levels was observed among these groups (data not shown). However, hepatic lesions occurred in both the control mice and the treated groups, and hepatic lesions were especially prevalent in mice treated with rapamycin alone (Supplementary Figure S5A & S5B). Hepatic toxicity, reflected by ALT and AST levels, was not observed in untreated mice or mice treated with metformin, cisplatin, or metformin plus cisplatin (Supplementary Figure S5C & S5D).

### Metformin activated AMPK and inhibited mTOR signaling *in vitro* and *in vivo*

**Figure 4:**
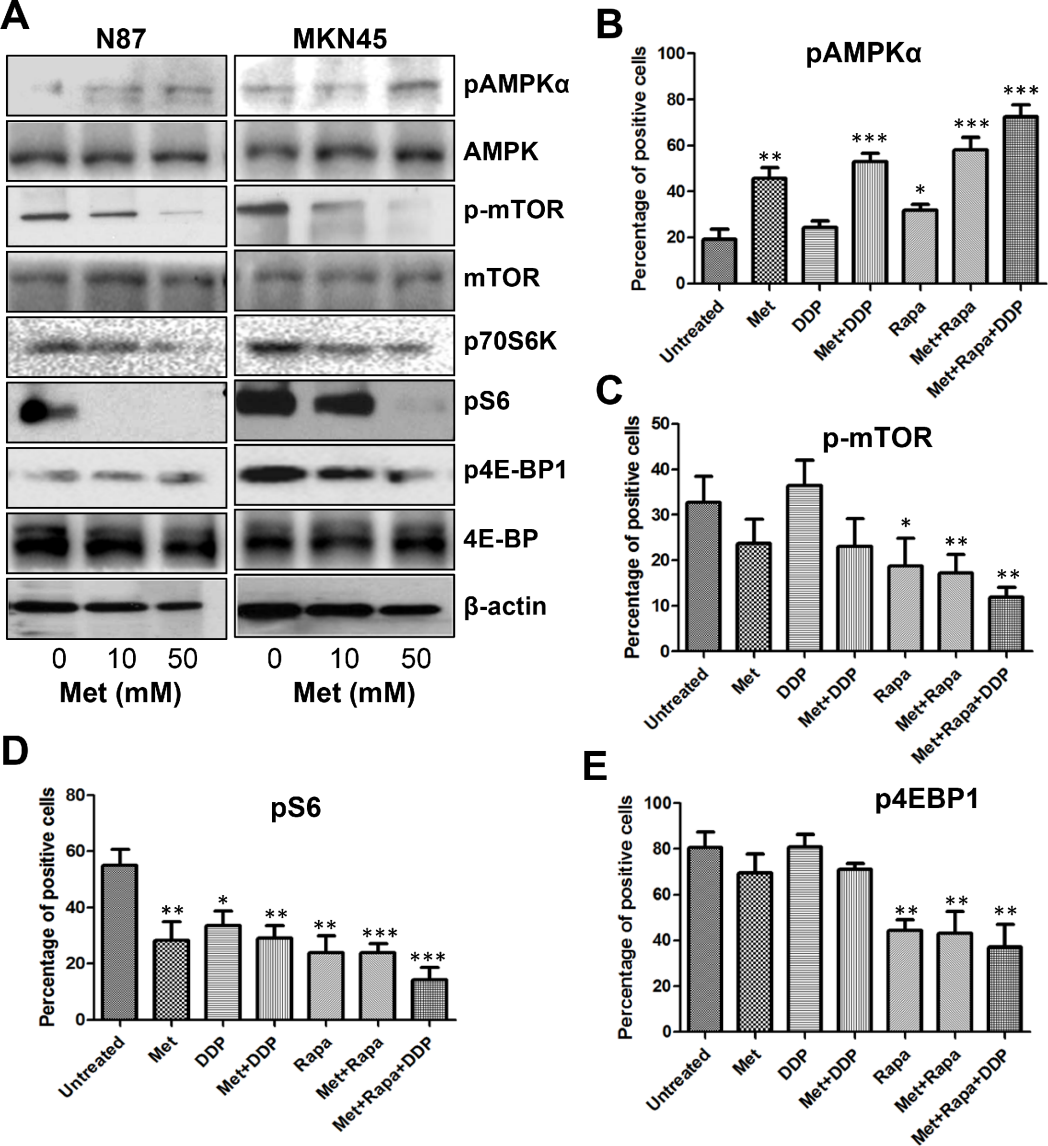
Metformin activated AMPKα and inhibited mTOR signaling in GC cells and MKN45 cell xenografts **(A)** GC cells were treated with different concentrations of metformin for 48 h and protein lysates were subjected to Western blot analysis for p-AMPKα, p-mTOR, p70S6K, p-S6, and p4EBP1; **(B–E)** Percentage of positive cells for pAMPKα **(B)**, p-mTOR **(C)**, pS6 **(D)**, and p4EBP1 **(E)** counted from five HPFs (high power field) (400×) per section from each mouse in each group. *P < 0.05; **P < 0.01, ***P < 0.001 compared with that in the untreated group (unpaired *t* test).

Metformin has been shown to exert anti-proliferative effects by activating AMPK which, in turn, suppresses the activity of mTOR signaling. Therefore, we investigated whether AMPK activation was induced in metformin-treated cells by detecting the levels of phophorylated-AMPK at Thr172. Treatment of metformin activated AMPK in a dose-dependent manner in both N87 and MKN45 cell lines (Figure 4A). These results were confirmed in xenograft tumor sections, which also showed a significant increase of pAMPKα expression after metformin treatment (Supplementary Figure S6).

To further confirm metformin regulation of the AMPK signaling pathways, we analyzed the phosphorylation status of mTOR and its downstream targets S6, 4EBP1, and P70S6K. Metformin decreased the phosphorylation of mTOR in a dose-dependent manner and also reduced the phosphorylation of S6, 4EBP1, and P70S6K (Figure 4A). Consistent with the results *in vitro*, the intensity and percentage of p-mTOR, pS6, and p4EBP1 staining of tumor sections was lower in the metformin treated group than in the untreated group (Supplementary Figure S6). These data confirmed that metformin treatment was a potent inhibitor of the mTOR pathway.

Although the combination of metformin with cisplatin, rapamycin, or both further increased pAMPKα expression and decreased p-mTOR and pS6 expression, cisplatin alone slightly stimulated pAMPKα and reduced pS6 expression levels, and no effect on p-mTOR and p4EBP1, while only a minimal effect on p4EBP1 expression (Figure 4B–4E, Supplementary Figure S7). Collectively, these data suggested that combining metformin with cisplatin, rapamycin, or both effectively decreased tumor burden by suppressing the pivotal AMPK/mTOR/S6 signaling axis.

### Sustained treatment of metformin led to aberrant expression of invasion/migration-related genes

**Figure 5:**
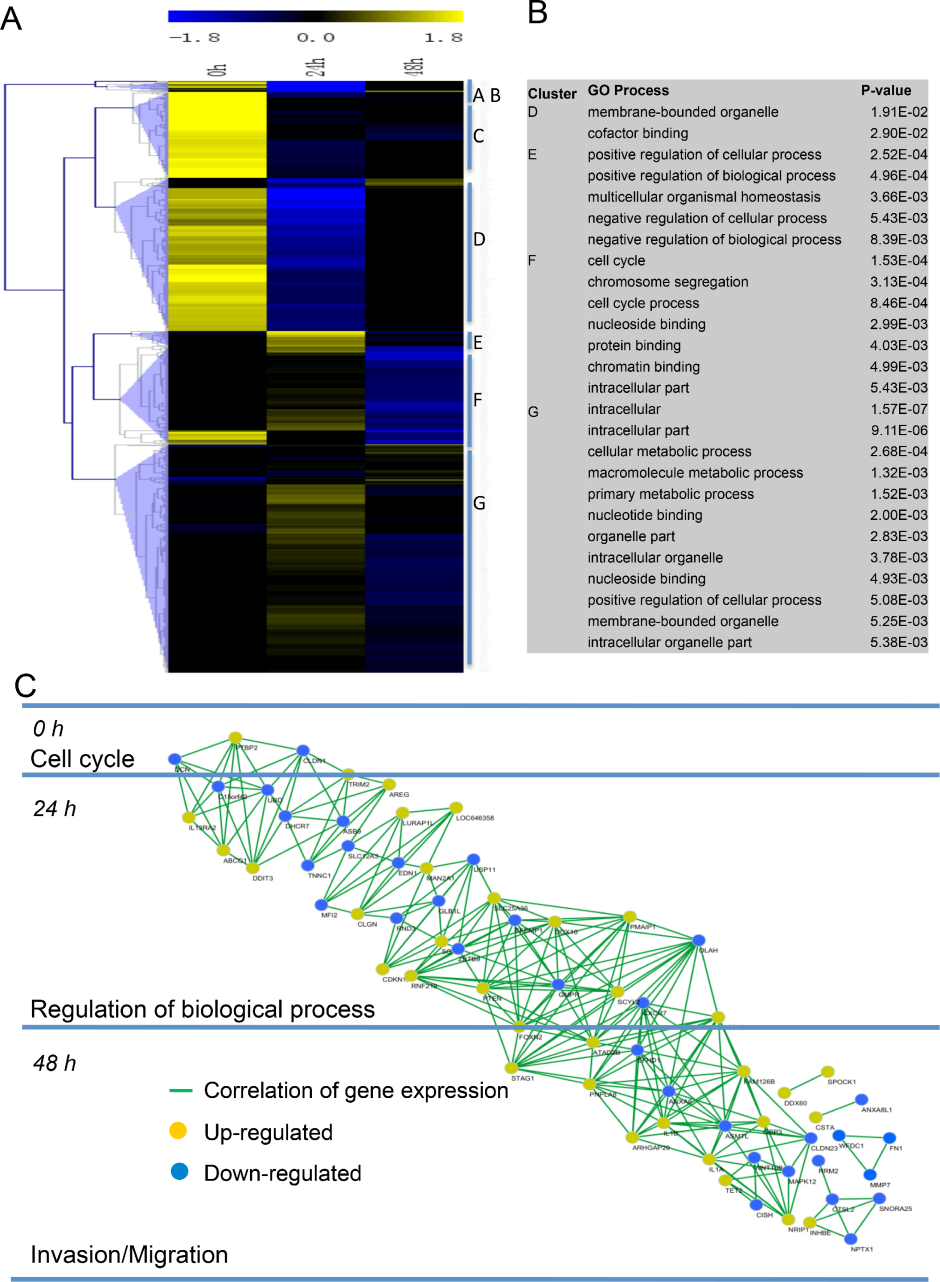
Metformin treatment led to aberrant expression of invasion/migration-related genes **(A)** Heatmap representing hierarchical clustering of all genes that displayed a 1.5-fold or greater difference in transcript levels in metformin-treated BGC823 cells with a concentration of 20 mM compared to controls at days 0, 24 h, and 48 h **(B)** Significant gene ontology (GO) terms retrieved by clusters D–G. **(C)** Pearson correlation network of the metformin action course generated using the Spring-embedded algorithm in Cytoscape. Partial genes in enriched GO categories are represented in the network. Nodes represent genes, and connections represent correlation coefficient.

In a global gene expression analysis, we found that 2025 genes were differentially expressed (1092 up-regulated and 933 down-regulated) at 24 h, while 503 were differentially expressed (280 up-regulated and 223 down-regulated) at 48 h (Figure 5A). These genes were clustered by expression pattern, yield several distinct clusters (such as, cell cycle, regulation of cellular process, regulation of biological process, metabolic process, *etc.*) (Figure 5B). According to gene expression patterns changed in the enriched GO categories, we created a network diagram to illustrate how these genes work over time by computing a Pearson correlation coefficient of all differentially expressed genes (Figure 5C). Our further analysis revealed that PTEN, a key inhibitor of Akt/mTOR pathway [20, 21], and CDKN1, a potent cyclin-dependent kinase inhibitor [22], were up-regulated at early stage after treated by metformin. However, genes associated with matrix degradation and tumor invasion displayed lower expression when treated with metformin persistently. These genes included DCN, CLDN1, FN1, MMP7, WFDC1, and UBD [23]. Strikingly, we observed that mRNA levels of DCN, MMP7, and WFDC1 didn't changed compared to control at earlier stage (24 h), but sharply reduced at later stage (48 h). Together, these data provided new evidences that sustained treatment of metformin leads to continuous damage of the ability of cell proliferation and invasion/migration.

**Figure 6:**
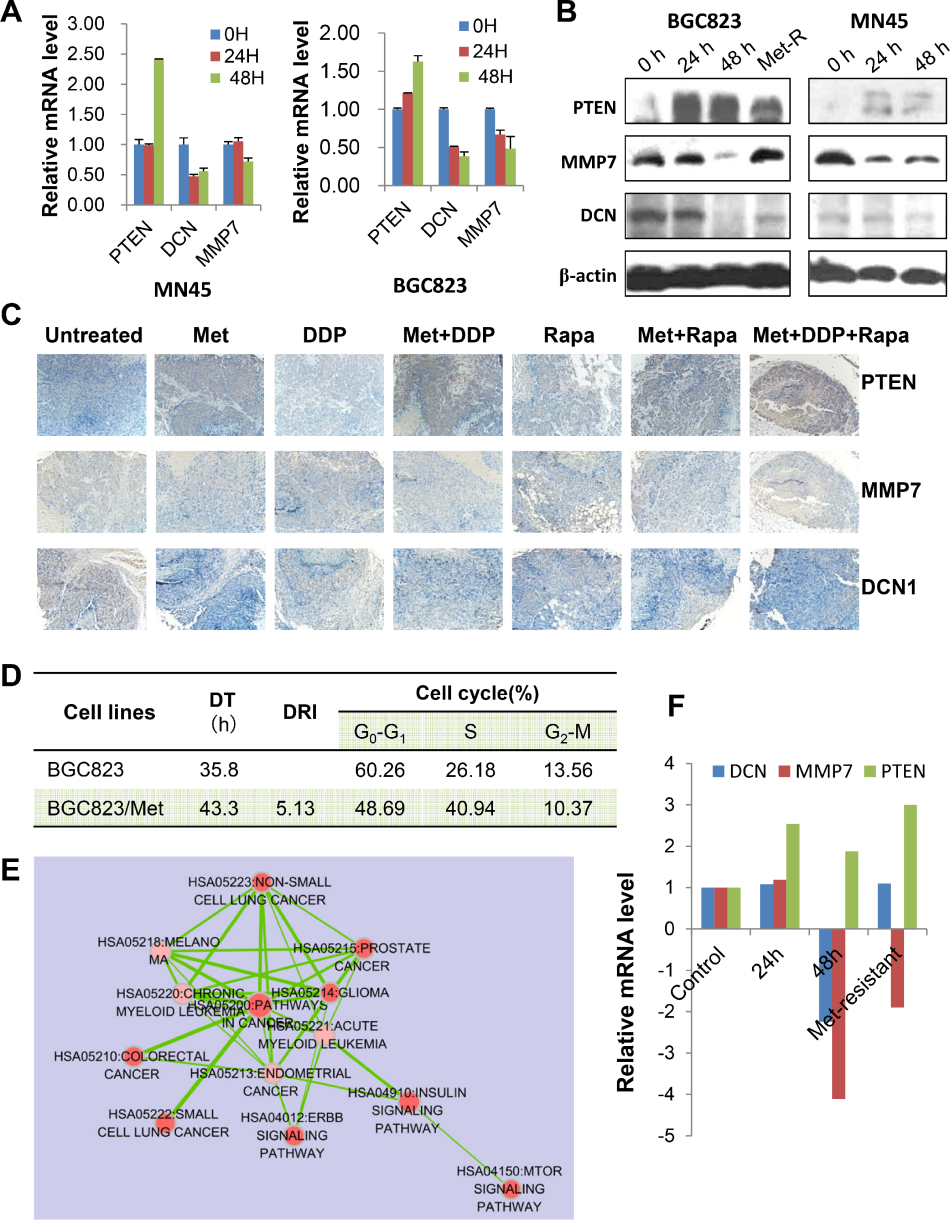
PTEN, DCN, and MMP7 expression in GC cells and the xenograft tumor samples **(A)** MNK45 and BGC823 cells were treated with metformin (20 mM) at indicated time points and mRNA levels were analyzed for PTEN, DCN and MMP7 by Real-time RT PCR. **(B)** Both BGC832 and MN45 cells were treated with metformin (20 mM) for indicated times. Metformin-resistant BGC823 (“Met-R”) cells were generated and used as a control. PTEN, DCN and MMP7 protein levels were determined using Western blotting. **(C)** Representative images of immunostaining for PTEN, DCN and MMP7 in tumor sections from each treatment group (IHC × 200). **(D)** The characteristics of metformin-resistant BGC823 cells (BGC823/Met). DT: double time; DRI: drug-resistant index. **(E)** Pathway analysis of all genes that displayed a 1.5-fold or greater difference in transcript levels in BGC823/Met cells compared to the parent cells. **(F)** The mRNA levels of PTEN, DCN and MMP7 in BGC823/Met cells, as well as BGC823 cells treated with metformin (20 mM) at 0 h, 24 h, and 48 h.

Next, we selected PTEN, DCN, and MMP and validated their association with metformin treatment in gastric cancer cells. Levels of PTEN mRNA and protein were increased when treated with metformin, while levels of MMP7 and DCN mRNA and protein were decreased (Figure 6A & 6B). These results were also confirmed by immunohistochemical analysis on the xenograft tumor samples, which showed an elevated expression of PTEN and decreased expression of MMP7 and DCN in metformin treated groups (Figure 6C).

Furthermore, little is known about the mechanisms underlying metformin-resistance of cancer cells. In this study, we generated a metformin-resistant gastric cancer cells through continuous incubation of BGC823 cells with high-concentrations of metformin. The characteristics of BGC823/Metformin were shown in Figure 6D. We found 5784 differentially expressed genes (4449 up-regulated and 1335 down-regulated) in metformin-resistant cells compared to parental cells. Pathway analysis revealed that mTOR pathway, ERBB pathway, and key pathways correlated with metformin-resistance (Figure 6E). We then focused on MMP7, DCN, and PTEN. PTEN mRNA level remained high level, but DCN and MMP7 mRNA levels went down as compared with that at 48 h (Figure 6F). Therefore, long-term and high-dose use of metformin reversed the expression of MMP7 and DCN, not PTEN.

## DISCUSSION

In the present study, we evaluated the effectiveness of metformin treatment alone or combination with the other commonly used therapeutic drugs, cisplatin and rapamycin, in gastric cancer, and determined the mechanism of metformin action. We demonstrated that treatment strategies containing metformin significantly reduced tumor growth and metastasis in nude mice, and that metformin caused an increase in AMPK and PTEN activity and suppression of mTOR/p70S6K/S6, as well as invasion/migration-associated genes, *e.g.*, MMP7, DCN and FN1. These results implicated the critically inhibitory roles of metformin in cell proliferation, tumor growth and metastasis in gastric cancer.

Researchers have widely studied p-mTOR, pS6, p4EBP1, PTEN, MMP7, and DCN expression in human solid cancer tissues, including gastric cancer. Key proteins in mTOR pathway and MMP7 expressions were unfavorable prognostic factors for gastric cancer [19, 24], while PTEN expression was a favorable prognostic factor [25]. In the present study with a small cohort of patients, we observed a higher expression of p-mTOR, pS6, p4EBP1, PTEN, and MMP7 proteins in primary gastric cancer tissues than in matched noncancerous tissues. Additionally, activation of AMPK and PTEN could inhibit mTOR signaling, implying that pAMPK and PTEN function as tumor suppressors in the development of human cancer. Our data showed a lower expression of pAMPK and PTEN in primary gastric cancer specimens than in the adjacent noncancerous mucosa. More clinical studies are needed to prove the predictive role of the above proteins in gastric cancer treated with AMPK agonists alone or in combination with mTORC1 inhibitors, such as rapamycin. In this study, we evaluated the therapeutic effect of a combination of metformin, an activator of AMPK, and rapamycin plus cisplatin in gastric cancer xenografts in mice.

Multiple retrospective studies have reported that metformin treatment was associated with a reduced risk for cancer and cancer-related mortality in diabetic populations. Lee *et al*. [18] reported that metformin reduced the incidence of several gastroenterological cancers in patients undergoing treatment for diabetes. In patients with diabetes not receiving treatment, cancer incidence increased two-fold for total, colorectal, and hepatic cancer. However, when patients were treated with metformin, the total, colorectal and hepatic cancer incidences decreased to near non-diabetic levels. Other studies in glioma, breast cancer and colon cancer models have shown that metformin effectively inhibited cell growth *in vitro* and significantly decreased the tumor burden *in vivo* [26–30]. Kim *et al.* [31] revealed that duration of metformin use was associated with the reduction in gastric cancer risk in type 2 diabetics without insulin treatment. Our results provided novel evidence that gastric cancer patients with 2DM receiving metformin treatment have longer survival duration than those without metformin treatment. This result is further supported by two newly published studies, which have confirmed the effect of metformin on gastric cancer [32, 33]. However, a multiple-center, larger cohort was needed to substantiate these results further. Previous data indicated that metformin exerted its growth inhibitory effects mainly by activating AMPK, which then suppressed the activity of mTOR and subsequently decreased its downstream effectors. In addition to suppressing the pivotal AMPK/mTOR/P70S6K axis, metformin has also been shown to modulate several other targets, including p53, p21, Cyclin D1, survivin and other cancer-related tyrosine kinase receptors such as HER2 [34, 35].

However, the metformin mechanism of action in gastric cancer has not been fully elucidated. A previous report by Kato *et al.* demonstrated that metformin treatment inhibited gastric cancer cell proliferation *in vitro* and *in vivo* by blocking the cell cycle with decreased expression of Cyclin D1 [36]. Our results demonstrated that cell growth and colony formation was inhibited with metformin treatment in a dose-dependent manner, and this correlated with decreased expression of Cyclin D1 and CDK4. Metformin also induced AMPK activation, leading to inhibition of the mTOR pathway, as indicated by decreased phosphorylation of S6 and its downstream effector, 4EBP1. These results were further confirmed in an animal model, which also showed that metformin effectively inhibited *in vivo* tumor burden of subcutaneous gastric cancer xenografts, although no significant difference was observed between mice treated with metformin alone and control mice. This may have been due to the relatively small number of mice in each group (*n* = 5) and the inherent characteristics of tumor cells used.

Given the fact that tumor growth was fully inhibited by metformin treatment alone in animal models, the combination of metformin with other drugs or inhibitors of this pathway may be an alternative strategy. Metformin has been shown to enhance cisplatin cytotoxicity *in vitro* and *in vivo* in various cancers. Rattan *et al.* [37] reported that metformin significantly increased cisplatin-induced cytotoxicity resulting in approximately 90% reduction in ovarian tumor growth. Gotlieb *et al.* [38] reported that metformin significantly inhibited the growth of ovarian cancer cell lines and enhanced the effect of cisplatin by inducing AMPK phosphorylation and decreasing p70S6K and S6K phosphorylation. Jiralerspong *et al.* [39] reported that diabetic patients with breast cancer taking metformin and undergoing neoadjuvant chemotherapy had a three-fold higher pathologic complete response rate than diabetics not taking metformin. Our data showed a significant inhibition of gastric cancer peritoneal dissemination when both agents were given via intraperitoneal injection. Interestingly, cisplatin resulted in a sharp weight loss, while its combination with metformin reduced this side effect. Harhaji-Trajkovic *et al.* [40] showed that cisplatin treatment triggered activation of AMPK and subsequent suppression of mTOR activity in glioma cells. Our data showed a slight increase of pAMPKα and a significant decrease of pS6, but no changes in p-mTOR and p4EBP1 when cisplatin was given. The effect of metformin on these proteins was not additive with cisplatin, suggesting that the enhanced anti-proliferation effect of this combination is due to the augmentation of cytotoxicity of both agents.

Since metformin alone or combination with cisplatin could not totally decrease proliferation of gastric cancer cells as determined by a relatively high expression of p-mTOR and p4EBP1, synchronous inhibition of mTOR signaling may be important for improving treatment efficiency in gastric cancer. Therefore, a treatment regimen containing rapamycin, a potent inhibitor of the mTOR pathway, may be necessary. Rapamycin treatment inhibits cell proliferation and tumor growth by blocking mTOR signaling. The results of this study show that when rapamycin is combined with metformin or metformin and cisplatin, the anti-proliferative effect was enhanced significantly *in vivo*, along with increased expression of pAMPKα and decreased expression of p-mTOR and pS6. However, severe hepatic toxicity was observed in mice treated with rapamycin alone. Considering that untreated mice also had hepatic lesions, this side effect may have been due to tumor burden along with rapamycin treatment. Hepatic lesions were less severe in mice treated with metformin, although an understanding of the underlying mechanisms will require further study.

In addition to the known targets of metformin, we have discovered that metformin treatment resulted in remarkable changes of genes expression patterns involved in cell cycles, metabolic and biological process, and invasion/migration. Among these altered genes, we found that PTEN responded significantly to metformin treatment. PTEN functions as a tumor suppressor by negatively regulating AKT/PKB signaling pathway. In present study, metformin increased the expression levels of PTEN mRNA and protein *in vivo* and *in vitro*, especially in metformin-resistant cancer cells, which makes PTEN an effective downstream target of metformin [41]. The most encouraging findings of our study is that metformin treatment led to decreased expression of several genes involved in tumor invasion and migration, including DCN, MMP7, FN1, and WFDC. DCN contains one attached glycosaminoglycan chain and plays a role in matrix assembly. DCN is capable of suppressing the growth and metastasis of various tumor cell lines [42]. Fibronectin (FN1) is involved in cell adhesion and migration processes [43]. MMP7, a member of the matrix metalloproteinase (MMP) family, is involved in the breakdown of extracellular matrix in disease processes, such as arthritis and metastasis [44]. cDNA array analysis at various time points suggested that metformin decreased the expression level of MMP7 and FN1 at a relatively later stage, indicating the usage of metformin must be continuously given. However, when tumor cells became resistant to metformin after long-term and high-dose inducement by metformin, MMP7 and DCN expression was restored again, implying that the dosage of metformin should be limited in a low level. Given the fact that the dose of metformin used *in vitro* is much higher than that inferred from the plasma levels achieved by oral metformin intake in human subjects, our data suggested that continuous use of metformin in lower-dose might be an alternative strategy for gastric cancer patients with 2DM. This notion is evidently supported by a recent study, showing that the increased cumulative 6 months duration of metformin use decreased the recurrence, all-cause mortality, and cancer-specific mortality rates among GC patients with diabetes [32].

In summary, the results from this study provided novel evidences that metformin could inhibit cell proliferation and tumor growth through targeting multiple key genes involved tumor proliferation, growth and metastasis, notably PTEN/Akt/mTOR pathway. PTEN and AMPK proteins were frequently inactivated and p-mTOR, pS6, p4EBP1, and MMP7 proteins were frequently downregulated in human gastric cancer. Therefore, our data provided novel evidence that the use of metformin as a therapeutic drug in combination with other inhibitors and standard chemotherapeutic agents could suppress cell proliferation, tumor growth, protein synthesis, and nodal metastasis.

## MATERIALS AND METHODS

### Human tissue specimens and patient information

Two cohorts of patients with gastric cancer were enrolled in this study. Firstly, we screened more than 2, 000 gastric cancer patients treated at the Fuzhou General Hospital and found seventy-four patients with type 2 diabetes (T2DM). Among these 74 cases, 22 cases (29.7%) were metformin user, the other 52 (70.3%) were non-metformin users. Of these patients, 50 (67.6%) were male and 24 (32.4%) were female; 37 (50.0%) were under the age of 66 years and 37 (50.0%) were 66 years old or greater with a median age of 66 years; 10 (13.5%) tumors were of tumor-node-metastasis stage (TNM) I, 21 of (28.4%) TNM stage II, and 43(58.1%) of TNM stage III. Clinical follow-ups were available for all these patients (median, 17 mo [range, 1–71 mo]) (Supplementary Table S1). The other cohort contains 39 patients with primary gastric adenocarcinoma underwent curative surgery at Changhai Hospital (Shanghai, China) and their tissues analyzed for the expression profiles of investigated proteins. None of these patients underwent preoperative chemotherapy and/or radiation therapy. In this cohort, patients consisted of 25 (64.1%) men and 14 (35.9%) women; 22 (56.4%) were under the age of 60 years and 17 (43.6%) were 60 years of age or greater with a mean age of 59.5 years. Thirty-seven patients (94.9%) presented with adenocarcinoma and two patients (5.1%) with mucinous adenocarcinoma. Of these, seven (17.9%) tumors were of tumor-node-metastasis stage (TNM) I, five of (12.8%) TNM stage II, 20 (51.3%) of TNM stage III, and seven (17.9%) of TNM stage IV. All patients were followed up for more than five years. Clinical follow-up results were available for all patients. All tissue specimens were obtained for this study with informed consent, and the use of human specimens was approved by the Changzheng and Changhai Hospital Institutional Review Board.

### Reagents and antibodies

Metformin was purchased from Sangon Biotechnology (Shanghai, China). Rapamycin was purchased from Gene Operation (MI, USA). Antibodies to phospho-ACC (pACC, Ser79), phospho-AMPKα (pAMPKα, Thr172), phospho-mTOR (p-mTOR, Ser2448), phospho-S6 (pS6, Ser235/236), phospho-4EBP1 (p4EBP1, Thr37/46), and Cyclin D1 (92G2) were purchased from Cell Signaling Technology (Beverly, MA, USA). PTEN (G6), MMP7 (L-17), DCN1 (N-15), and anti-β-actin were from Santa Cruz Biotechnology (Dallas, TX, USA).

### Cell lines and culture conditions

The human gastric cancer cell lines, AGS, N87, MKN28, MGC803, BGC823, HGC27, and MKN45, were purchased from the Cell Center of Chinese Academy of Sciences, Shanghai, China. N87 cells were maintained in RPMI1640 with 10% fetal bovine serum (FBS) (Invitrogen Corp., Grand Island, NY, USA), AGS cells were maintained in F12 with 10% FBS, and the other cells were maintained in DMEM with 10% FBS. The cell lines were cultured in a 37°C humidified atmosphere containing 95% air and 5% CO_2_.

### Cell proliferation assay

Cells were trypsinized, counted, and seeded into 96-well plates at a density of 5, 000 cells per well. Twenty-four hours after seeding, cells were treated with metformin at concentrations of 0, 10, 20, or 50 mM. At 0, 24, and 48 h after metformin treatment, cell proliferation was measured using the CCK8 assay (Dojindo Kumamoto, Japan). The percentages of surviving cells at 48 h relative to survival at 0 h were calculated. The experiment was repeated three times independently.

### Colony formation assay

Cells were seeded in 6-well plates in triplicate at a density of 500 cells per well (N87 cells) or 200 cells per well (MKN45 cells). After 24 h, cells were treated with metformin (0, 10, 50 mM) for 14 days. The colonies were fixed with methanol/acetone (1:1) and stained with crystal violet. Colonies with more than 50 cells per colony were counted.

### Flow cytometric analysis

Flow cytometric analysis was performed to determine the effects of metformin on cell cycle distribution. Briefly, gastric cancer cells, grown in 6-well plates (2 × 10^5^ cells/well), were treated with metformin (0, 20, 50 mM) for 48 hours. Then, cells were harvested by trypsinization and fixed with 70% ethanol, and measured following the manufacturer's protocol (KEY GEN, Nanjing, China). Cell cycle distribution was analyzed by flow cytometry (FACSCalibur, BD Biosciences, Bedford, MA).

### Xenograft model of human gastric cancer

Subcutaneous tumor xenograft models were used to assess the treatment effect of metformin alone. MKN45 cells (1 × 10^6^ cells in 0.1mL PBS) were injected subcutaneously into the right flank of 4-week-old male Balb/c nude mice. The animals were randomized into control and treatment groups (*n* = 5). Metformin treatment (250 mg/kg) was initiated when tumors reached a mean diameter of 4 mm and was given once daily by intraperitoneal injection. The control group received saline only. Tumor diameter was measured every two or three days. At 15 days, all animals were sacrificed and the tumors collected. Tumor volume (mm^3^) was calculated as *V* = 0.52 (length × width × depth).

Peritoneal tumor xenograft models were constructed to assess the treatment effect of metformin alone or in combination with rapamycin or cisplatin. MKN45 cells (1 × 10^6^ cells) were inoculated into the intraperitoneal cavity of mice. Treatment with metformin (250 mg/kg, *i.p., q.d.*) with or without rapamycin (2.5 mg/kg, *i.p., q.d.)* was started 10 days after cell inoculation (day 0). Cisplatin treatment (4 mg/kg, *i.p.*) was given on days 0, 7, and 14 along with metformin treatment. Animals were sacrificed 14 days after treatment. All animal experiments were approved by the Animal Ethics Committee of the Second Military Medical University.

### cDNA microarray analysis

BGC823 cells were treated with metformin at a concentration of 20 mM for 24 h and 48 h and then total RNA from these cells was extracted using TRIZOL^®^ Reagent (Invitrogen life technologies). RNA purity and integrity was determined by the NanoDrop^®^ ND-1000 and denaturing agarose gel electrophoresis. Gene expression profiling was performed using AFFEMERIX (AFFY U219). Target preparation and RNA reversion and amplification were performed according to the manufacturer's instruction. For ratio calculation, we obtained the average of signal intensities of Cy3:Cy5 of each spot and the ratio > 2 or < 0.5 was defined as the cut-off benchmark to determine the up-regulated or down-regulated genes. Gene ontology analysis was used to the most valuable genes as previously described [45, 46].

### Real-time RT-PCR

Real-time RT-PCR of 3 selected genes (PTEN, MMP7, and FN1) was carried out using by SYBR Premix Ex Taq (Perfect real-time) kit (Takara) in the Rotor Gene 3000 system (Corbet Research, Sydney, Australia). GAPDH was used as the internal control. Relative mRNA abundance was calculated as 2^−ΔCt^ [ΔCt = Ct (target gene)-Ct (GAPDH)]. The primers used for real-time PCR are listed in Supplementary Table S3.

### Western blot analysis

Standard western blotting was done as previously described [19]. Briefly, whole-cell lysates were prepared from N87 and MKN45 cells at the indicated times after treatment. Cell lysates were resolved by SDS/PAGE and transferred electrophoretically to PVDF membrane (Bio-Rad Lab., Hercules, CA, USA). The membranes were probed with specific antibodies and the immunoreactive proteins were detected by the enhanced chemiluminescene (ECL) kit (Santa Cruz, CA, USA).

### Immunohistochemistry

Consecutive tissue sections (4 μm) of paraffin-embedded normal and tumor specimens were prepared and processed for immunohistochemical analysis as described previously [47, 48]. Antibodies against pAMPKα (1:100), pACC (1:200), p-mTOR (1:50), pS6 (1:100), p4EBP1 (1:200), PTEN (1:100), MMP7 (1:100), and FN1 (1:150) were used to determine protein expression. Sections were scored blindly by two independent individuals using an Olympus CX31 microscope (Olympus Optical). The following values for scoring intensity were used: 0, absence of positive staining; 1, weak expression; 2, moderate expression; 3, strong expression. A mean percentage of positive tumor cells were determined in at least five fields of view at 400x magnification and assigned *a* value from 0 to 100%. The percentage of positive tumor cells and the staining intensity were multiplied to produce a weighted score for each case. The scores ranged from 0 (0% of cells staining) to 3 (100 × 3/100).

### Statistical analysis

Categorical data were analyzed using chi-squared tests. The Kaplan-Meier method was used to estimate survival rates, and the log-rank test was used to assess survival differences between groups. The Cox proportional hazards model for multivariate survival analysis was used to assess predictors related to survival. The significance of the *in vitro* data was determined using a two-tailed Student's *t*-test, whereas significance of the *in vivo* data was determined using the two-tailed Mann-Whitney *U* test. Analyses were done using the SPSS statistical software program for Microsoft Windows. In all of the tests, a two-sided *P* < 0.05 was defined as statistically significant [19].

## SUPPLEMENTARY FIGURES AND TABLES



## Abbreviations

AMPK: AMP-activated protein kinase
Met: Metformin
Rapa: Rapamycin
Cisplatin: DDP

## References

[1] Nakahara K, Tsuruta O, Tateishi H, Arima N, Takeda J, Toyonaga A, Sata M (2004). Extended indication criteria for endoscopic mucosal resection of early gastric cancer with special reference to lymph node metastasis—examination by multivariate analysis. Kurume Med J.

[2] Sakuramoto K, Okajima K, Iga C, Nishimura J, Toyoda M, Kawashima Y (1989). [A case of advanced gastric cancer with Virchow's node metastasis, responding to concomitant plasma exchange and immunochemotherapy]. Gan To Kagaku Ryoho.

[3] Sowa M, Kato Y, Nishimura M, Kubo T, Maekawa H, Umeyama K (1989). Surgical approach to early gastric cancer with lymph node metastasis. World J Surg.

[4] Yoshida A, Imamura A, Tanaka H, Hirano M, Kamma H, Ueno E, Ushio H, Aiyoshi Y, Soeda S (1989). A case of metastasis from gastric cancer to the thyroid gland. Jpn J Surg.

[5] Yoshida K, Ohta K, Ohhashi I, Nakajima T, Takagi K, Nishi M (1988). [Studies on gastric lymphatics by using activated carbon particle (CH44) and lymph node metastasis of gastric cancer]. Nihon Geka Gakkai Zasshi.

[6] Vakana E, Altman JK, Platanias LC (2012). Targeting AMPK in the treatment of malignancies. J Cell Biochem.

[7] Hardie DG, Ross FA, Hawley SA (2012). AMP-Activated Protein Kinase: A Target for Drugs both Ancient and Modern. Chem Biol.

[8] Hardie DG (2011). AMP-activated protein kinase: a cellular energy sensor with a key role in metabolic disorders and in cancer. Biochem Soc Trans.

[9] Habu H, Takeshita K, Sunagawa M, Endo M (1986). Lymph node metastasis in early gastric cancer. Int Surg.

[10] Choudhury Y, Yang Z, Ahmad I, Nixon C, Salt IP, Leung HY (2014). AMP-activated protein kinase (AMPK) as a potential therapeutic target independent of PI3K/Akt signaling in prostate cancer. Oncoscience.

[11] Yoshikawa K, Kitaoka H (1984). Clinicopathologic studies of gastric cancer with metastasis to the liver—based on the cases detected at initial surgery. Jpn J Clin Oncol.

[12] Koay E, Sulman EP (2012). Management of brain metastasis: past lessons, modern management, and future considerations. Curr Oncol Rep.

[13] Saydah SH, Loria CM, Eberhardt MS, Brancati FL (2003). Abnormal glucose tolerance and the risk of cancer death in the United States. American journal of epidemiology.

[14] Michels KB, Solomon CG, Hu FB, Rosner BA, Hankinson SE, Colditz GA, Manson JE (2003). Nurses’ Health S. Type 2 diabetes and subsequent incidence of breast cancer in the Nurses’ Health Study. Diabetes care.

[15] Micic D, Cvijovic G, Trajkovic V, Duntas LH, Polovina S (2011). Metformin: its emerging role in oncology. Hormones (Athens).

[16] Pulito C, Sanli T, Rana P, Muti P, Blandino G, Strano S (2013). Metformin: On Ongoing Journey across Diabetes, Cancer Therapy and Prevention. Metabolites.

[17] Pandey A, Forte V, Abdallah M, Alickaj A, Mahmud S, Asad S, McFarlane SI (2011). Diabetes mellitus and the risk of cancer. Minerva endocrinologica.

[18] Lee MS, Hsu CC, Wahlqvist ML, Tsai HN, Chang YH, Huang YC (2011). Type 2 diabetes increases and metformin reduces total, colorectal, liver and pancreatic cancer incidences in Taiwanese: a representative population prospective cohort study of 800, 000 individuals. BMC cancer.

[19] Yu G, Wang J, Chen Y, Wang X, Pan J, Li G, Jia Z, Li Q, Yao JC, Xie K (2009). Overexpression of phosphorylated mammalian target of rapamycin predicts lymph node metastasis and prognosis of chinese patients with gastric cancer. Clin Cancer Res.

[20] Chalhoub N, Baker SJ (2009). PTEN, and the PI3-kinase pathway in cancer. Annual review of pathology.

[21] Renner O, Blanco-Aparicio C, Carnero A (2008). Genetic modelling of the PTEN/AKT pathway in cancer research. Clin Transl Oncol.

[22] Tang L, Ling X, Liu W, Das GM, Li F (2012). Transcriptional inhibition of p21WAF1/CIP1 gene (CDKN1) expression by survivin is at least partially p53-dependent: evidence for survivin acting as a transcription factor or co-factor. Biochem Biophys Res Commun.

[23] Zhao S, Jiang T, Tang H, Cui F, Guo F, Liu C, Lu H, Xue Y, Jiang W, Peng Z (2014). Ubiquitin D is an Independent Prognostic Marker for Survival in Stage IIB-IIC Colon Cancer Patients Treated with 5-Fluoruracil-Based Adjuvant Chemotherapy. J Gastroenterol Hepatol.

[24] Fanelli MF, Chinen LT, Begnami MD, Costa WL, Fregnami JH, Soares FA, Montagnini AL (2012). The influence of transforming growth factor-alpha, cyclooxygenase-2, matrix metalloproteinase (MMP)-7, MMP-9 and CXCR4 proteins involved in epithelial-mesenchymal transition on overall survival of patients with gastric cancer. Histopathology.

[25] Lee HS, Lee HK, Kim HS, Yang HK, Kim WH (2003). Tumour suppressor gene expression correlates with gastric cancer prognosis. J Pathol.

[26] Soritau O, Tomuleasa C, Aldea M, Petrushev B, Susman S, Gheban D, Ioani H, Cosis A, Brie I, Irimie A (2011). Metformin plus temozolomide-based chemotherapy as adjuvant treatment for WHO grade III and IV malignant gliomas. J BUON.

[27] Wang YY, Zhang W, Qian S, Liu R, Kan ZX, Wang JH (2012). The effect of locoregional transarterial infusion chemotherapy on liver metastasis after gastric cancer resection. J Int Med Res.

[28] Gonzalez-Angulo AM, Meric-Bernstam F (2010). Metformin: a therapeutic opportunity in breast cancer. Clin Cancer Res.

[29] Hosono K, Endo H, Takahashi H, Sugiyama M, Sakai E, Uchiyama T, Suzuki K, Iida H, Sakamoto Y, Yoneda K (2010). Metformin suppresses colorectal aberrant crypt foci in a short-term clinical trial. Cancer Prev Res (Phila).

[30] Bodmer M, Meier C, Krahenbuhl S, Jick SS, Meier CR (2010). Long-term metformin use is associated with decreased risk of breast cancer. Diabetes Care.

[31] Kim YI, Kim SY, Cho SJ, Park JH, Choi IJ, Lee YJ, Lee EK, Kook MC, Kim CG, Ryu KW (2014). Long-term metformin use reduces gastric cancer risk in type 2 diabetics without insulin treatment: a nationwide cohort study. Alimentary pharmacology & therapeutics.

[32] Lee CK, Jung M, Jung I, Heo SJ, Jeong YH, An JY, Kim HI, Cheong JH, Hyung WJ, Noh SH (2015). Cumulative Metformin Use and Its Impact on Survival in Gastric Cancer Patients After Gastrectomy. Annals of surgery.

[33] Greenhill C (2015). Gastric cancer: Metformin improves survival and recurrence rate in patients with diabetes and gastric cancer. Nature reviews Gastroenterology & hepatology.

[34] Bost F, Sahra IB, Le Marchand-Brustel Y, Tanti JF (2012). Metformin and cancer therapy. Curr Opin Oncol.

[35] Martin-Castillo B, Vazquez-Martin A, Oliveras-Ferraros C, Menendez JA (2010). Metformin and cancer: doses, mechanisms and the dandelion and hormetic phenomena. Cell Cycle.

[36] Kato K, Gong J, Iwama H, Kitanaka A, Tani J, Miyoshi H, Nomura K, Mimura S, Kobayashi M, Aritomo Y (2012). The antidiabetic drug metformin inhibits gastric cancer cell proliferation *in vitro* and *in vivo*. Molecular cancer therapeutics.

[37] Rattan R, Graham RP, Maguire JL, Giri S, Shridhar V (2011). Metformin suppresses ovarian cancer growth and metastasis with enhancement of cisplatin cytotoxicity *in vivo*. Neoplasia.

[38] Iranikhah M, Wilborn TW, Wensel TM, Ferrell JB (2012). Denosumab for the prevention of skeletal-related events in patients with bone metastasis from solid tumor. Pharmacotherapy.

[39] Jiralerspong S, Palla SL, Giordano SH, Meric-Bernstam F, Liedtke C, Barnett CM, Hsu L, Hung MC, Hortobagyi GN, Gonzalez-Angulo AM (2009). Metformin and pathologic complete responses to neoadjuvant chemotherapy in diabetic patients with breast cancer. J Clin Oncol.

[40] Harhaji-Trajkovic L, Vilimanovich U, Kravic-Stevovic T, Bumbasirevic V, Trajkovic V (2009). AMPK-mediated autophagy inhibits apoptosis in cisplatin-treated tumour cells. J Cell Mol Med.

[41] Lin F, Yan W, Wen T, Wu GY (2013). [Metformin induces apoptosis in hepatocellular carcinoma Huh-7 cells *in vitro* and its mechanism]. Zhonghua Zhong Liu Za Zhi.

[42] Shintani K, Matsumine A, Kusuzaki K, Morikawa J, Matsubara T, Wakabayashi T, Araki K, Satonaka H, Wakabayashi H, Iino T (2008). Decorin suppresses lung metastases of murine osteosarcoma. Oncol Rep.

[43] Knowles LM, Gurski LA, Engel C, Gnarra JR, Maranchie JK, Pilch J (2013). Integrin alphavbeta3 and fibronectin upregulate Slug in cancer cells to promote clot invasion and metastasis. Cancer Res.

[44] Fukuda A, Wang SC, Morris JPt, Folias AE, Liou A, Kim GE, Akira S, Boucher KM, Firpo MA, Mulvihill SJ (2011). Stat3 and MMP7 contribute to pancreatic ductal adenocarcinoma initiation and progression. Cancer Cell.

[45] Yu GZ, Chen Y, Long YQ, Dong D, Mu XL, Wang JJ (2008). New insight into the key proteins and pathways involved in the metastasis of colorectal carcinoma. Oncol Rep.

[46] Klattenhoff CA, Scheuermann JC, Surface LE, Bradley RK, Fields PA, Steinhauser ML, Ding H, Butty VL, Torrey L, Haas S (2013). Braveheart, a long noncoding RNA required for cardiovascular lineage commitment. Cell.

[47] Wang J, Wang LP, Xu S, Yang GZ (2013). Morphology, immunohistochemistry and hTERC gene in-situ hybridization in Barrett's esophagus]. Zhonghua Bing Li Xue Za Zhi.

[48] Yu G, Wang J, Chen Y, Wang X, Pan J, Li Q, Xie K (2008). Tissue microarray analysis reveals strong clinical evidence for a close association between loss of annexin A1 expression and nodal metastasis in gastric cancer. Clin Exp Metastasis.

