# Supplementary material for: Metformin potentiates rapamycin and cisplatin in gastric cancer in mice
Source: Oncotarget. 2015 Feb 28;6(14):12748–62. doi: 10.18632/oncotarget.3327 (PMC4494971; doi:10.18632/oncotarget.3327)
Supplement: Supplementary file 1 [file oncotarget-06-12748-s001.pdf]

SUPPLEMENTARY FIGURES AND TABLES

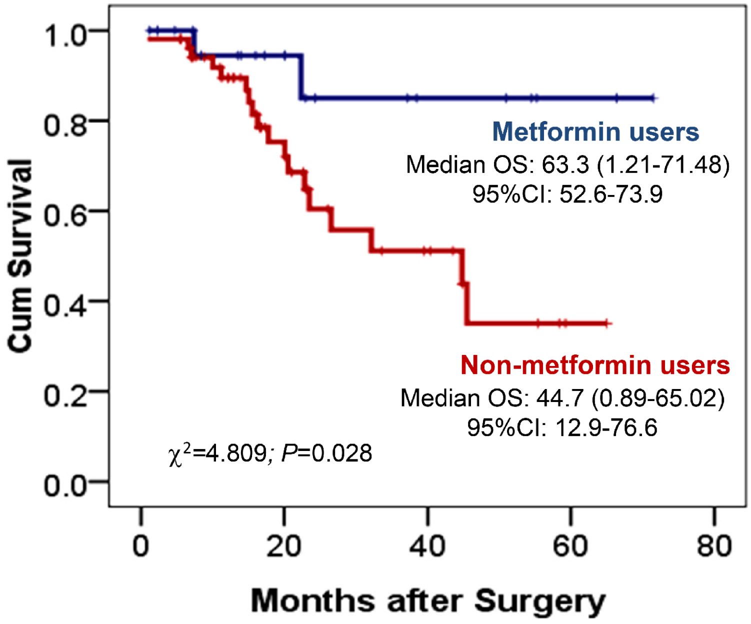

Num. at risk (mon)	0	20	40	60	80
Metformin users	22	12	4	4	0
Non-metformin users	52	22	8	1	0

Supplementary Figure S1: Patients with both gastric cancer and type 2 diabetics who used metformin (metformin user) showed a relatively longer survival duration than those who did not use metformin (non-metformin user) (*P* = 0.028).

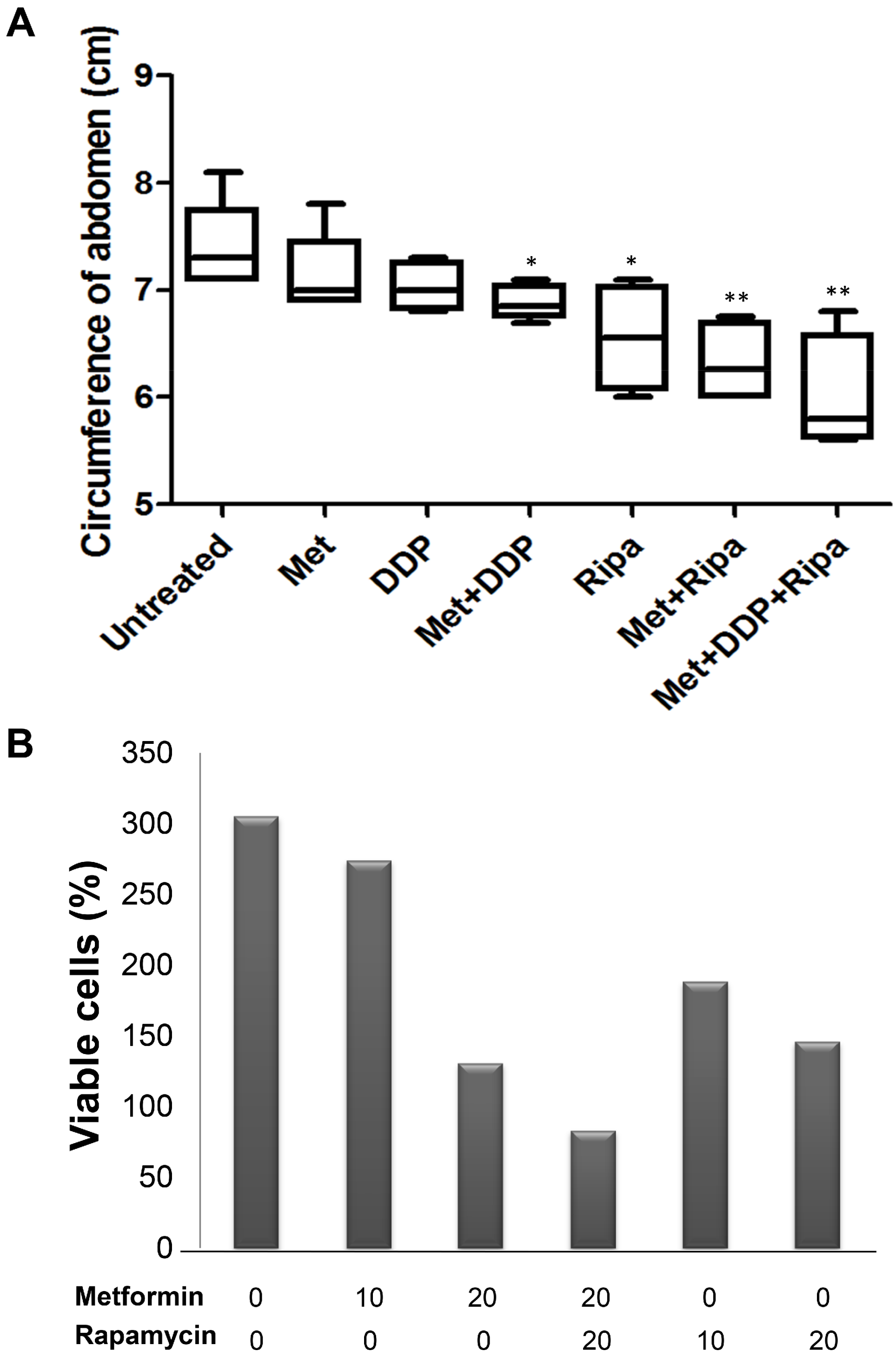

Supplementary Figure S2: (A) Peritoneal tumor xenograft models. MKN45 cells (1 × 10^6 cells) were inoculated into the intraperitoneal cavity of mice. Treatment with metformin (250 mg/kg, *i.p., q.d.*) with or without rapamycin (2.5 mg/kg, *i.p., q.d.*) was started 10 days after cell inoculation (day 0). Cisplatin treatment (4 mg/kg, i.p.) was given on days 0, 7, and 14 along with metformin treatment. Animals were sacrificed 14 days after treatment. Abdominal circumferences of mice were measured. *$P < 0.05$; **$P < 0.01$, ***$P < 0.001$ compared with that in the untreated group (unpaired T test). (B) Cell proliferation. MKN45 cells were trypsinized, counted, and seeded into 6-well plates at a density of 5,000 cells per well. Twenty-four hours after seeding, cells were treated with metformin at concentrations of 0, 10, or 20 mM. At 0 h and 48 h after metformin treatment, cell proliferation was measured using the CCK8 assay. The percentages of surviving cells at 48 h relative to survival at 0 h were calculated. The experiment was performed three times independently with similar results.

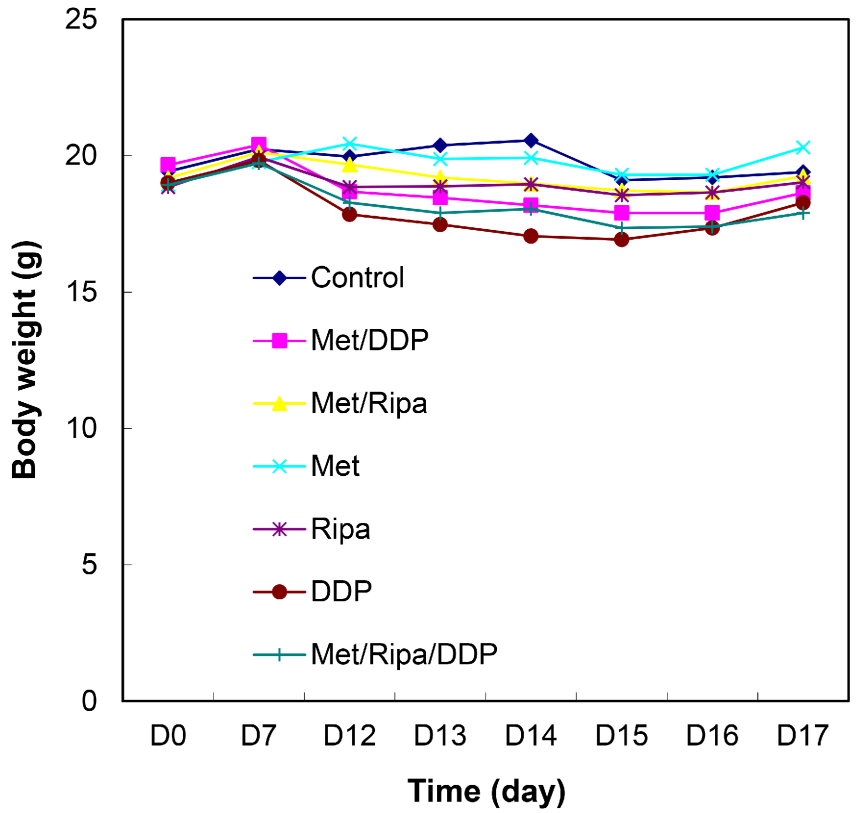

Supplementary Figure S3: Body weight of mice implanted with peritoneal tumors and treated with metformin, cisplatin, rapamycin or a combination of treatments. MKN45 cells (1 × 10^6 cells) were inoculated into the intraperitoneal cavity of mice. Treatment with metformin (250 mg/kg, *i.p., q.d.*) with or without rapamycin (2.5 mg/kg, *i.p., q.d.*) was started 10 days after cell inoculation (day 0). Cisplatin treatment (4 mg/kg, *i.p.*) was given on days 0, 7, and 14 along with metformin treatment. Animals were sacrificed 14 days after treatment. Body weights of mice were measured.

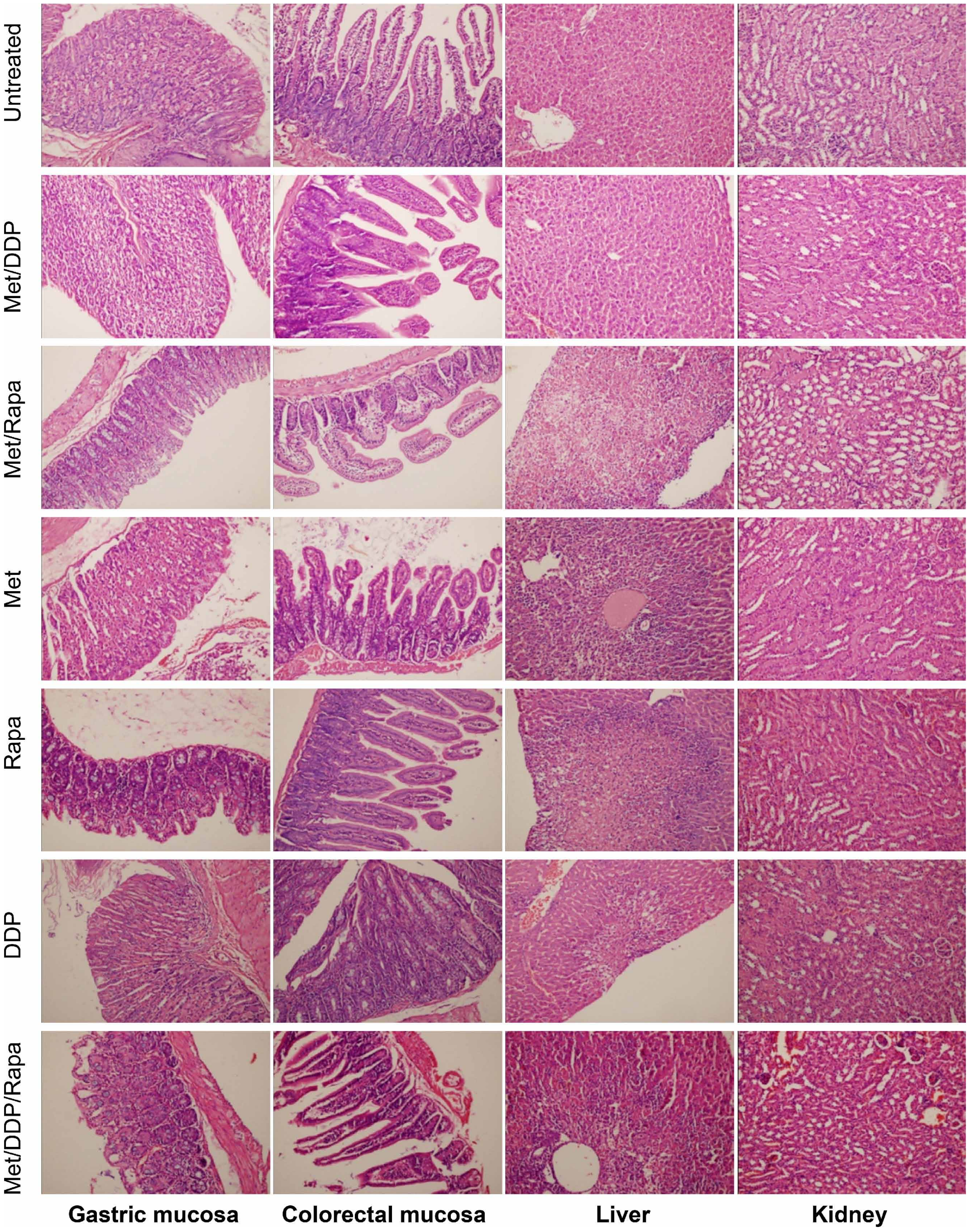

Supplementary Figure S4: H&E stained sections of gastric, colorectal, and kidney tissues from each group. MKN45 cells (1×10^6 cells) were inoculated into the intraperitoneal cavity of mice. Treatment with metformin (250 mg/kg, *i.p., q.d.*) with or without rapamycin (2.5 mg/kg, *i.p., q.d.*) was started 10 days after cell inoculation (day 0). Cisplatin treatment (4 mg/kg, *i.p.*) was given on days 0, 7, and 14 along with metformin treatment. Animals were sacrificed 14 days after treatment. Organs were collected and tissues were processed for pathological analysis. No obvious morphological changes were observed except in the liver (the arrow indicated the lesion).

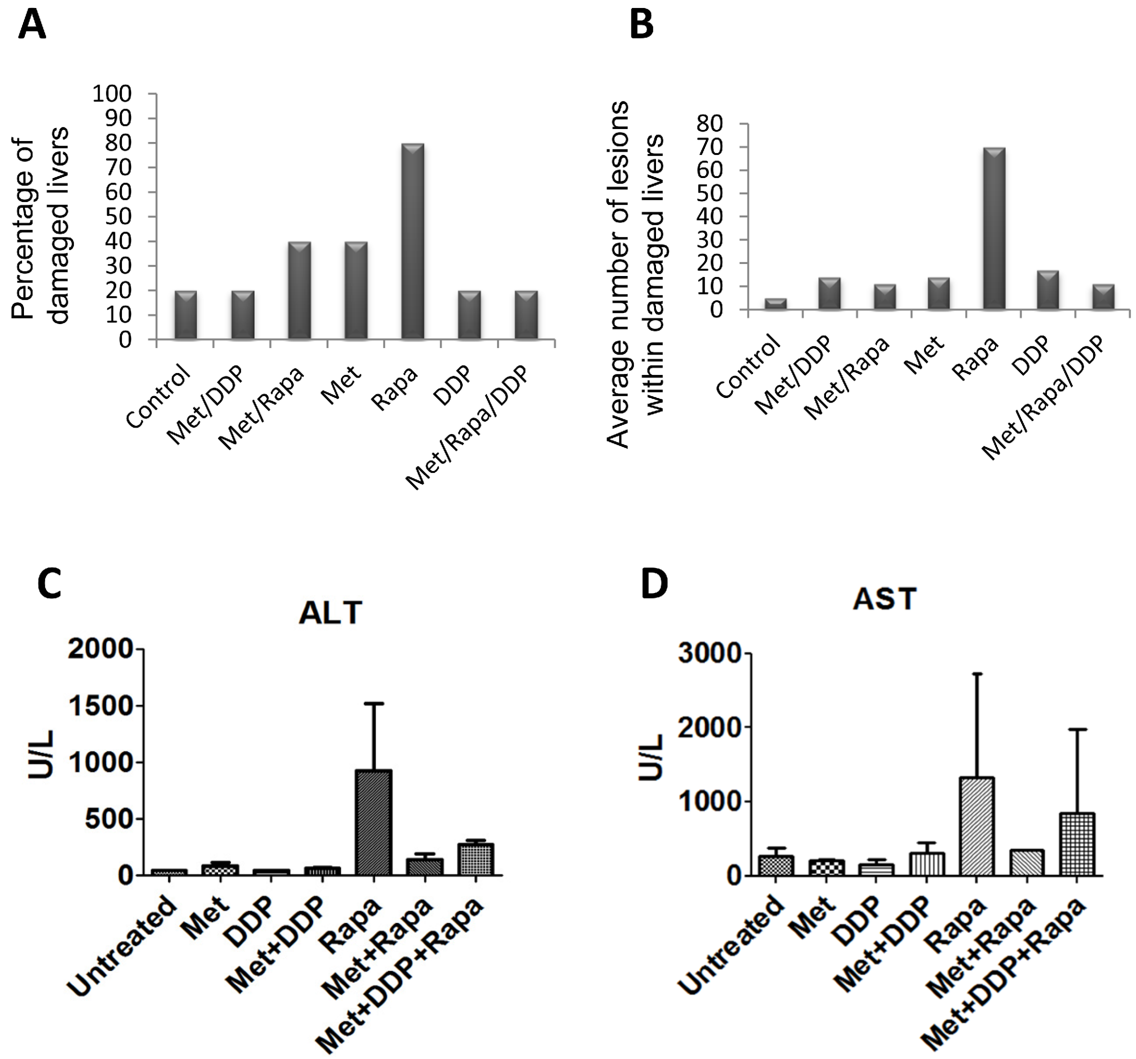

Supplementary Figure S5: Hepatic physiologic profile in plasma from mice implanted with intraperitoneal MKN45 cells and treated with inhibitors. **(A)** Average percentage of damaged livers in mice from each treatment group. **(B)** Average number of liver lesions in mice with damaged livers from each group. **(C)** ALT levels in each group. **(D)** AST levels in each group.

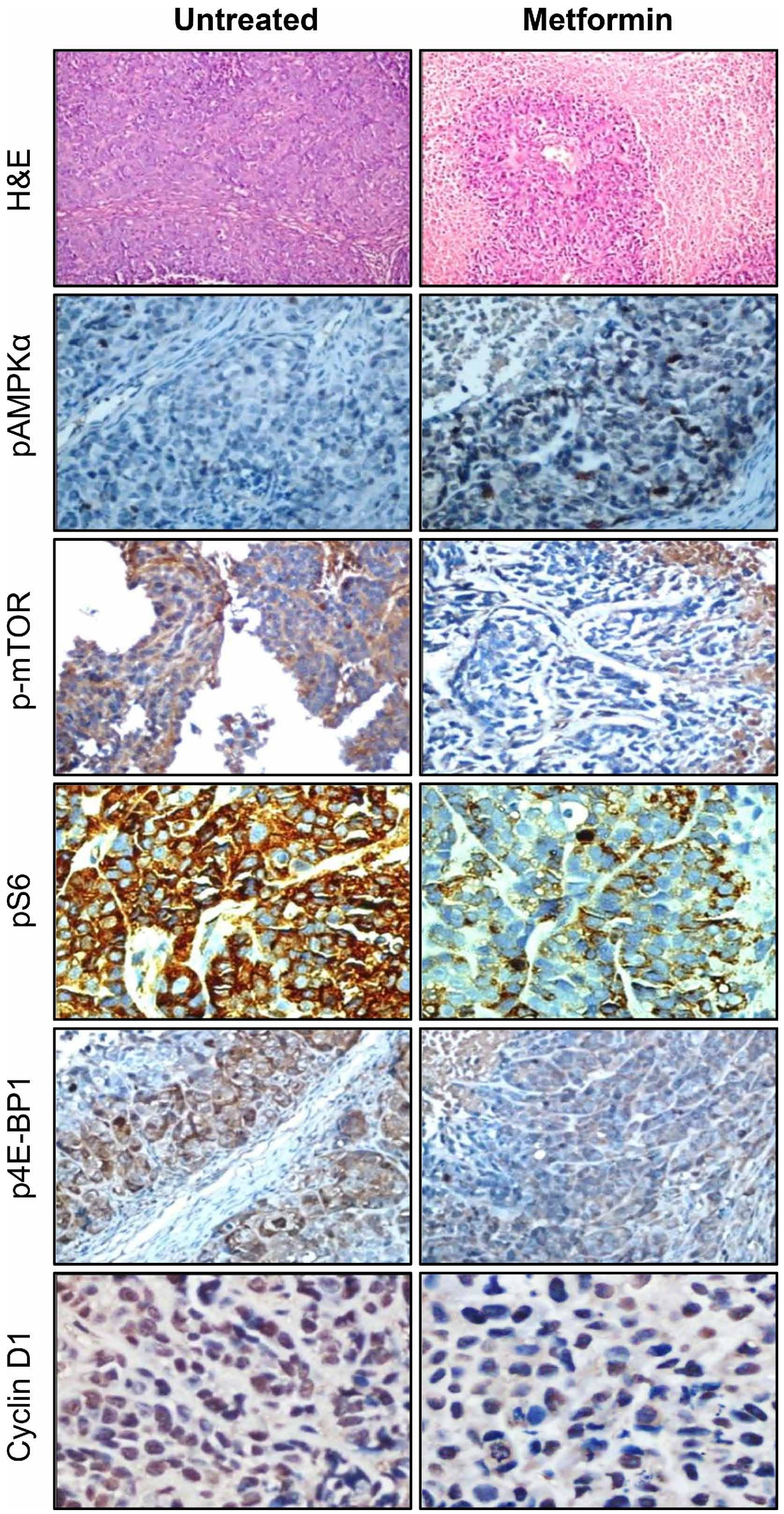

Supplementary Figure S6: Gene expression in tumor tissues mice received metformin treatment. MKN45 cells (1×10^7) were subcutaneously injected into the right flanks of female nude mice and treated with metformin (250 mg/kg) or NS (control) *i.p. q.d.* when the tumors reached a mean diameter of 4 mm. On day 15, mice were sacrificed and tumors were collected and tumor sections were stained for pAMPKα, p-mTOR, pS6, p4EBP1, and Cyclin D1 expression. The Representative images of immunostaining of tumor sections from metformin treated and untreated groups were shown.

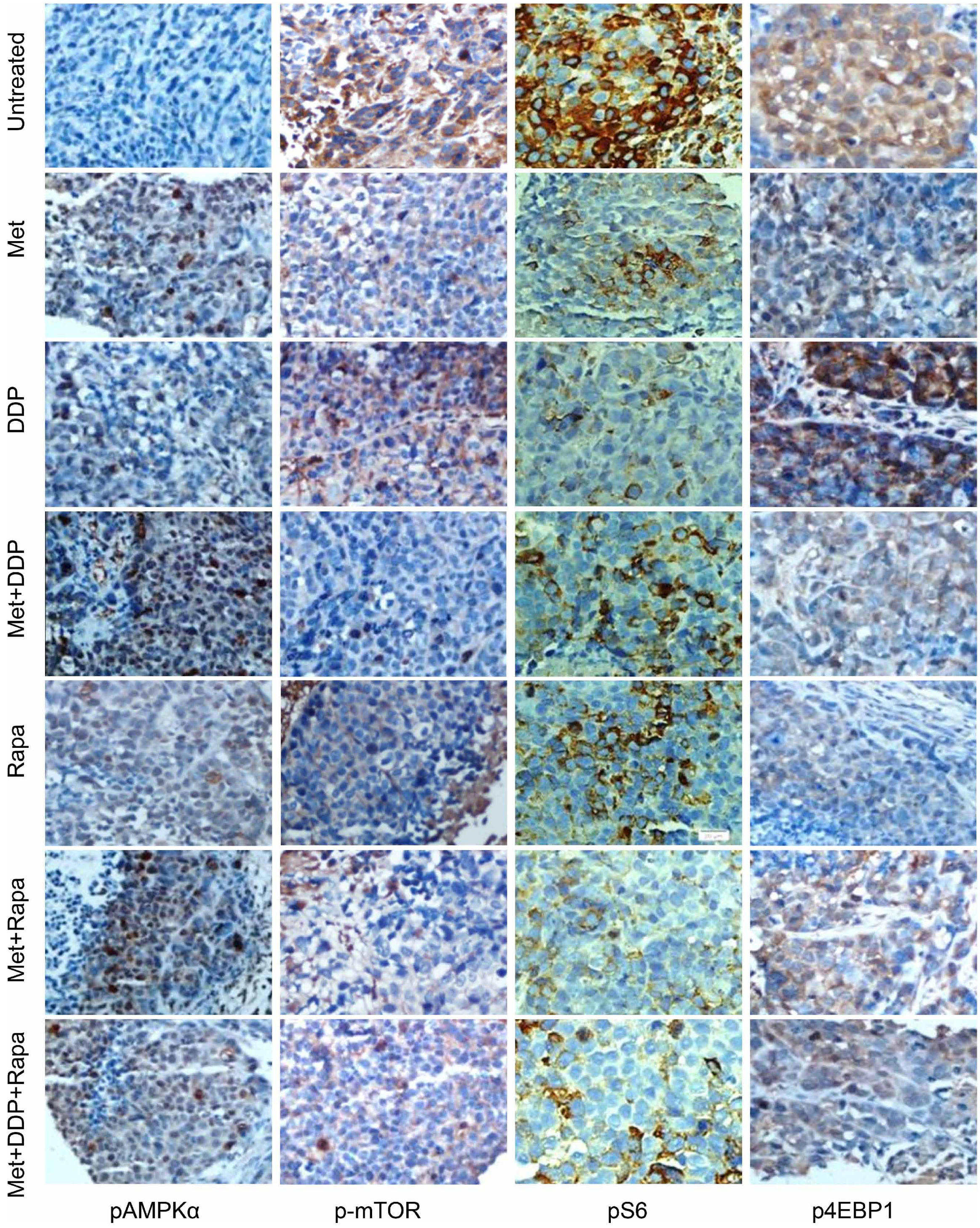

Supplementary Figure S7: Gene expression in tumor tissues mice received treatment of metformin, cisplatin or rapamycin, or both. MKN45 cells (1×10^6 cells) were inoculated into the intraperitoneal cavity of mice. Treatment with metformin (250 mg/kg, *i.p., q.d.*) with or without rapamycin (2.5 mg/kg, *i.p., q.d.*) was started 10 days after cell inoculation (day 0). Cisplatin treatment (4 mg/kg, *i.p.*) was given on days 0, 7, and 14 along with metformin treatment. Animals were sacrificed 14 days after treatment. Peritoneal tumors were collected and tumor sections were stained for the expression of pAMPKα, p-mTOR, pS6, and p4EBP1 proteins. Representative images of immunostaining of tumor sections from each treatment group were shown.

Supplementary Table S1: Detailed information of gastric cancer patients with 2DM

Variables	*N*	Metformin user (%)	Non-metformin users (%)
**Gender**			
Male	50	16 (32.0)	34 (68.0)
Female	24	6 (25.0)	18 (75.0)
**Age**			
<66y	37	15 (40.5)	22 (59.5)
≥66y	37	17 (45.9)	20 (54.1)
**Tumor Type**			
Adenocarcinoma	67	19 (28.4)	48 (71.6)
Mucinous Adenocarcinoma	4	2 (50.0)	2 (50.0)
Ring Cell Carcinoma	3	1 (33.3)	2 (66.7)
**Differentiation**			
Well/Moderate	68	21 (30.9)	47 (69.1)
Poor/Undifferentiated	6	1 (16.7)	5 (83.3)
**Tumor Stage**			
I/II	31	8 (25.8)	23 (74.2)
III	43	14 (32.6)	29 (67.4)

Supplementary Table S2: Cox regression model of a small cohort of gastric cancer patients with 2DM

Variables	No.	Mean Survival (months)	*P* (univariate)	*P* (multivariate)	HR	(95% CI)
**Gender**						
male	50	43.9	0.558			
female	24	57				
**Age**						
< 66y	37	55.2	0.042	0.066	0.394	(0.146 to 1.063)
≥ 66y	37	39.5				
**N stage**						
N0	20	57.1	0.057	0.233	0.395	(0.086 to 1.820)
N1–3	54	36.7				
**T stage**						
T1/T2	20	55.9	0.104			
T3/T4	54	38.9				
**Differentiation**			0.872			
Well/Moderate	68	47.1				
Poor/Undifferentiated	6	41.1				
**Tumor stage**			0.075	0.54	0.672	(0.189 to 2.389)
I/II	31	53.5				
III	43	37.1				
**Metformin user**						
No	52	39.1	0.028	0.076	3.858	(0.868 to 17.154)
Yes	22	63.3				

Supplementary Table S3: Primer list of PTEN, DCN, and MMP7

No.	Gene Symbol	Gene Bank Access #	Forward primer	Reverse primer	Product length (bp)	Ta (°C)
1	PTEN	NM_000314.4	tgattcagcctcttcagatact	tctgattgggatgaggcatta	124	60
2	DCN	NM_133507.2	ctcagctatttcttctacctct	gtcaggaaatgtatgctttgtg	105	60
3	MMP7	NM_002423.3	ggatggtagcagtctaggg	catcactgcattaggatcagag	100	60
4	GAPDH	NM_017008.3	gcgagatcccgctaacatca	ctcgtggttcacacccatca	178	60
